# *The Organon* and Materia Medica Club of the Bay Cities of California

**Published:** 1896-08

**Authors:** W. E. Ledyard

**Affiliations:** Secretary


W. E. Ledyard, *Secretary.*

Reported by Eleanor F. Martin, M. D.

---

### *THE ORGANON* AND MATERIA MEDICA CLUB OF THE BAY CITIES OF CALIFORNIA.

The regular semi-monthly meeting was held at the office of Dr. George H. Martin, 606 Sutter Street, San Francisco, Friday evening, March 20th, 1896.

Members present: Drs. J. M. Selfridge, G. J. Augur, M. F. Underwood, M. T. Wilson, C. M. Selfridge, and George H. Martin.

The meeting was called to order at 8.10 o'clock by the President, Dr. J. M. Selfridge. The minutes of the previous meeting were read, and, after slight corrections by Drs. J. M. Selfridge and Underwood, were approved.

Dr. J. M. Selfridge then read from *The Organon*, Sections 74 and 75.

### Discussion.

Dr. J. M. Selfridge—I had a case of la grippe, in which the patient attempted to cure herself with Quinine, which resulted in an asthmatic condition, with considerable cough and inability to lie down. This continued for three weeks. There was one symptom that attracted my attention: *after sleeping, very profuse perspiration, which came on immediately upon waking.* I looked it up and found that there was only one remedy in the materia medica that had this symptom, and this remedy was *Lac-caninum.* I gave one dose of the CM potency, and she was better the next day. She had many symptoms of *Lac-caninum.*

Dr. Martin—It is strange how a single symptom will so often make the other symptoms clear.

Dr. J. M. Selfridge—I would rather depend on a single key-note symptom than a lot of uncertain symptoms.

Dr. Wilson—I think it a very safe method to prescribe for key-note symptoms.

Section 76 was read.

### Discussion.

Dr. J. M. Selfridge—Many cases are cured by prescribing for a single symptom.

Dr. Underwood—I have cured two cases with the following symptom: *Sensation as if there were a hole in the epigastric region going clear to the back, with sensation of air going through it.* *Rhus*$^{cm}$ cured one, and *Medorrhinum*$^{cm}$ the other.

Sections 77–80 were read.

### Discussion.

Dr. J. M. Selfridge—Homœopathy has been made fun of from the fact that allopaths claim that itch is due to a microbe. For this reason they make fun of Hahnemann's explanation of the itch. He states, however, that itch is only a symptom of some internal disease.

Dr. Underwood—Can all persons take the itch?

Dr. J. M. Selfridge—No. Some persons would have to be exposed a good deal in order to get it.

Dr. Underwood—According to the vaccination law, the person who has had it once is not likely to get it again.

Section 81 was read.

### Discussion.

Dr. Martin—We are called by our brethren of the other school the unscientific practitioners, but I think this name would be suitable for them in some of their treatment. For instance, the rest-cure for nervous prostration. The majority of allopathic physicians usually say that in order to carry out the rest-cure you should put your patient in a darkened room and keep her there for six weeks. During all this time they stuff the patient full of certain kinds of food, treating all patients alike. A number of patients are helped, perhaps, but many are made almost insane by the enforced quiet. This is not scientific treatment. They do not differentiate their cases.

Dr. J. M. Selfridge—Is there a distinction between nervous prostration and neurasthenia?

Dr. Martin—No.

Sections 82–95 were read.

### Discussion.

Dr. J. M. Selfridge—Case to illustrate a cure by key-note symptom. A lady who had been in the habit of wearing high-neck and long-sleeve dresses was invited to a party, where she wore low-neck and no sleeves. Asthma occurred. She went to Philadelphia, and I advised her to see Hering, which she did, but he did not cure her. She returned home and had repeated attacks. Finally she said to me, "*I feel as if wind were blowing upon my back.*" I gave her a few doses of *Hepar-sulphur* and cured her.

Dr. Martin—I had a case of a man with severe headaches. I studied his case very carefully, and relieved him some almost the first thing, but did not cure. He was satisfied, but I was not. I wanted to cure him. One day I was feeling of his head and discovered a round bald place, under long hair, with a peculiar eruption over the scalp. I questioned him regarding it, and he said that just before his headaches commenced a blister came on his head just where this bald spot was. He had had some fever and the next morning found this blister. It broke in two or three days, and the headaches then commenced. They were occipital pains, sometimes intense. I thought of *Sulphur*, and looked it up. I had given him *Belladonna*, *Gelsemium*, *Hyoscyamus*, etc. I found that *Sulphur* covered nearly all of his symptoms; gave it in the 200th potency, and his sister reported four months later that he felt so well that he thought it unnecessary to come again.

The meeting then adjourned to meet again the first Friday in

April, at Dr. Martin's office, in San Francisco, when the reading of *The Organon* would be commenced at Section 96.

